# Use of Augmented Reality for Training Assistance in Laparoscopic Surgery: Scoping Literature Review

**DOI:** 10.2196/58108

**Published:** 2025-01-28

**Authors:** Francisco Javier Celdrán, Javier Jiménez-Ruescas, Carlos Lobato, Lucía Salazar, Juan Alberto Sánchez-Margallo, Francisco M Sánchez-Margallo, Pascual González

**Affiliations:** 1 I3A LoUISE Research Group University of Castilla-La Mancha Albacete Spain; 2 Bioengineering and Health Technologies Unit Jesús Usón Minimally Invasive Surgery Centre Cáceres Spain; 3 Scientific Direction Jesús Usón Minimally Invasive Surgery Centre Cáceres Spain; 4 Computing Systems Department University of Castilla-La Mancha Albacete Spain

**Keywords:** laparoscopic surgery, surgical training, surgical simulator, augmented reality–based laparoscopic simulator, AR-based laparoscopic simulator, augmented reality, mobile phone

## Abstract

**Background:**

Laparoscopic surgery training is a demanding process requiring technical and nontechnical skills. Surgical training has evolved from traditional approaches to the use of immersive digital technologies such as virtual, augmented, and mixed reality. These technologies are now integral to laparoscopic surgery training.

**Objective:**

This scoping literature review aimed to analyze the current augmented reality (AR) solutions used in laparoscopic surgery training.

**Methods:**

Following the PRISMA-ScR (Preferred Reporting Items for Systematic Reviews and Meta-Analyses extension for Scoping Reviews) guidelines, we conducted a scoping review using 4 databases: Scopus, IEEE Xplore, PubMed, and ACM. Inclusion and exclusion criteria were applied to select relevant articles. Exclusion criteria were studies not using AR, not focused on laparoscopic surgery, not focused on training, written in a language other than English, or not providing relevant information on the topics studied. After selecting the articles, research questions (RQs) were formulated to guide the review. In total, 2 independent reviewers then extracted relevant data, and a descriptive analysis of the results was conducted.

**Results:**

Of 246 initial records, 172 (69.9%) remained after removing duplicates. After applying the exclusion criteria, 76 articles were selected, with 25 (33%) later excluded for not meeting quality standards, leaving 51 (67%) in the final review. Among the devices analyzed (RQ 1), AR video–based devices were the most prevalent (43/51, 84%). The most common information provided by AR devices (RQ 1) focused on task execution and patient-related data, both appearing in 20% (10/51) of studies. Regarding sensorization (RQ 2), most studies (46/51, 90%) incorporated some form of sensorized environment, with computer vision being the most used technology (21/46, 46%) and the trainee the most frequently sensorized element (41/51, 80%). Regarding training setups (RQ 3), 39% (20/51) of the studies used commercial simulators, and 51% (26/51) made use of artificial models. Concerning the evaluation methods (RQ 4), objective evaluation was the most used, featured in 71% (36/51) of the studies. Regarding tasks (RQ 5), 43% (22/51) of studies focused on full surgical procedures, whereas 57% (29/51) focused on simple training tasks, with suturing being the most common among the latter (11/29, 38%).

**Conclusions:**

This scoping review highlights the evolving role of AR technologies in laparoscopic surgery training, although the impact of optical see-through devices remains unclear due to their limited use. It underscores the potential of emerging technologies such as haptic feedback, computer vision, and eye tracking to further enhance laparoscopic skill acquisition. While most relevant articles from other databases were included, some studies may have been missed due to the specific databases and search strategies used. Moreover, the need for standardized evaluation metrics is emphasized, paving the way for future research into AR’s full potential in laparoscopic skill acquisition.

## Introduction

### Background

Training in laparoscopic surgery is a long and demanding process that requires extensive theoretical knowledge, along with technical and nontechnical skills. Learning models in surgical training have rapidly evolved from traditional approaches based on the educational philosophy of “*see one, do one, teach one*” to more sophisticated surgical simulators that aim to increase the number of training sessions, thus dramatically enhancing the skills of medical professionals and the safety of patients [1]. Among the formative strategies, there are some based on animal models [2] and cadavers [3]. However, due to the economic and ethical issues involved in some of these solutions, surgical training has rapidly shifted toward the use of simulation-based systems, mainly for the early formative stages [4].

The emergence of immersive digital technologies such as virtual reality, augmented reality (AR), and mixed reality (MR) has led to a paradigm shift in the field of surgical training. Virtual reality allows users to be immersed in a fully digital environment replacing the physical world, whereas AR superimposes virtual elements onto the real world, enhancing or augmenting the user’s environment. The latest evolution of these immersive technologies is MR, which merges virtual and real objects, enabling realistic interactions and coexistence. The use of these technologies is becoming an important part of the training process in laparoscopic surgery, enhancing the training experience and content without putting the patient at risk [5,6]. This technique can generate customized 3D models of each patient on which to train. Similarly, during the training process, this technology allows for the inclusion of enriched information, such as holographic images or 3D objects to guide the user during the training process and facilitating a more precise alignment between virtual information and physical objects in a simulator, an experimental model, or a patient. The use of AR technologies is constantly evolving and being integrated into the field of minimally invasive surgery. However, the current level of development of this technology as a tool to assist in the training process in laparoscopic surgery, as well as the available solutions (both commercial and prototypes) and the training functionalities they offer, is not precisely known.

### Objectives

This scoping review intended to analyze the current AR and MR solutions used to assist in laparoscopic surgery training. Similarly, we reviewed the types of surgical simulators that make use of this technology, the training assistance information offered, and the main training tasks and procedures in which they have been used. This gave us a more detailed view of the current state of AR in the field of laparoscopic surgery training.

## Methods

### Search Strategy

A structured bibliographical search was conducted in the Scopus, IEEE Xplore, PubMed, and ACM databases. The same search query adapted to each database query syntax was used. We used a set of keywords related to the topic of this scoping review to identify relevant studies published up to January 1, 2024 (Multimedia Appendix 1). In general terms, the initial search encompassed all articles that included the following terms in the title or abstract or as keywords: “laparoscopic”; AND “augmented reality” OR “mixed reality” OR “extended reality”; AND “training” OR “practice.”

### Eligibility Criteria

A series of inclusion and exclusion criteria were considered to select the articles that best applied for our objectives. In general, articles on studies that did not make use of AR technologies or did not provide information on the inclusion of such technologies in laparoscopic surgery training, articles that did not focus on laparoscopy or its skill training, articles that were written in a language other than English, reviews, or articles that were not accessible were discarded. In addition, regarding the quality of the articles, those studies that did not provide relevant information on the topics studied were excluded.

### Selection of Articles

Results were screened by 2 independent reviewers. In case of discrepancies between reviewers regarding the inclusion or noninclusion of an article, a third reviewer was consulted. The PRISMA-ScR (Preferred Reporting Items for Systematic Reviews and Meta-Analyses extension for Scoping Reviews) guidelines were followed to carry out this scoping review [7], including the completion of the corresponding checklist (Multimedia Appendix 2).

### Research Questions

The aim of this work was to analyze the studies published in scientific literature in relation to the use of AR in laparoscopic surgery training and analyze the relevant aspects that facilitate the development of new investigations in this field. Therefore, with this scoping review, we intended to answer the following research questions (RQs):

1. What type of devices and feedback are used for AR-based laparoscopic surgery training? (RQ 1)
2. What type of sensorization is used for AR-based laparoscopic surgery training? (RQ 2)
3. What type of simulator and setup is used for AR-based laparoscopic surgery training? (RQ 3)
4. What type of evaluation is used to assess skill acquisition in AR-based laparoscopic surgery training? (RQ 4)
5. What type of surgical tasks or procedures are used in AR-based laparoscopic surgery training? (RQ 5)

**Figure 1:**
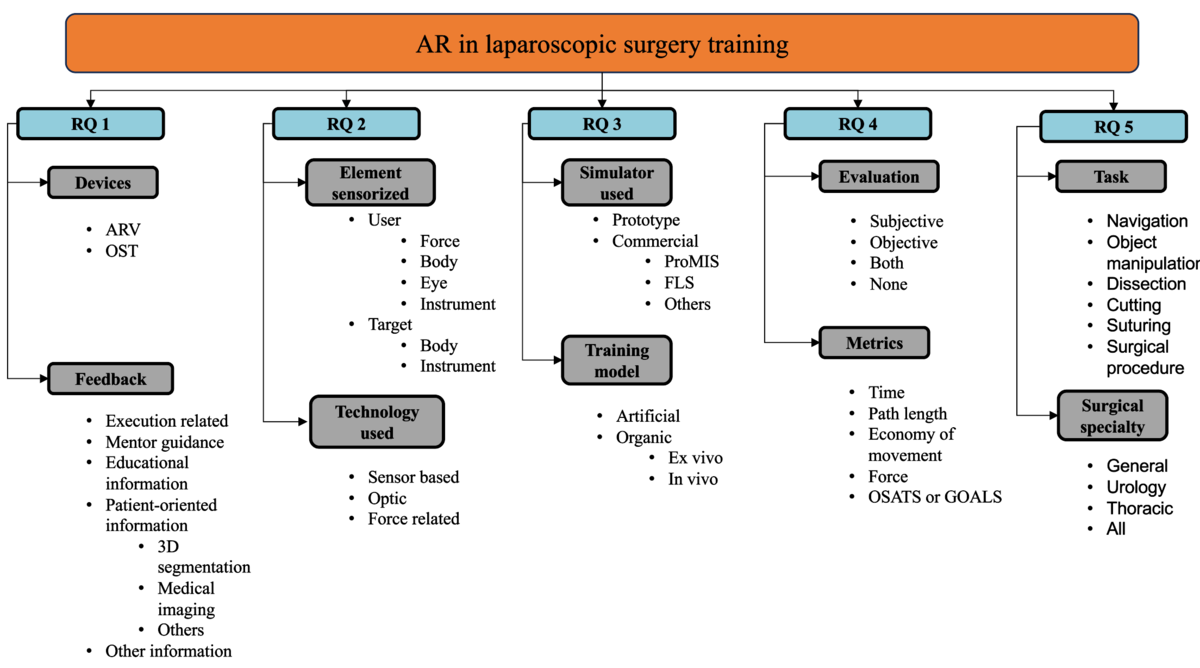
Taxonomy of articles regarding each research question (RQ). AR: augmented reality; ARV: augmented reality video; FLS: Fundamentals of Laparoscopic Surgery; GOALS: Global Operative Assessment of Laparoscopic Skills; OSATS: Objective Structured Assessment of Technical Skills; OST: optical see-through.

To do so, we classified every article based on different dimensions, each one related to one of the RQs (Figure 1).

### Data Collection and Processing

For each selected study, information on the following aspects was recorded: (1) year of publication, (2) summary of the complete study, (3) modality of teaching (tele-mentoring or conventional), (4) type of device used for AR, (5) type of information provided to the learner, (6) type of sensorization, (7) type of AR simulator (commercial or prototype, and which one if commercial), (8) type of evaluation (objective or subjective), (9) training tasks or procedures performed, and (10) specialty of laparoscopic surgery.

As with the selection of articles, the data collected for each article were screened by 2 independent reviewers. In case of discrepancies between reviewers regarding the data collected for each article, a third reviewer was consulted.

## Results

### Overview

**Figure 2:**
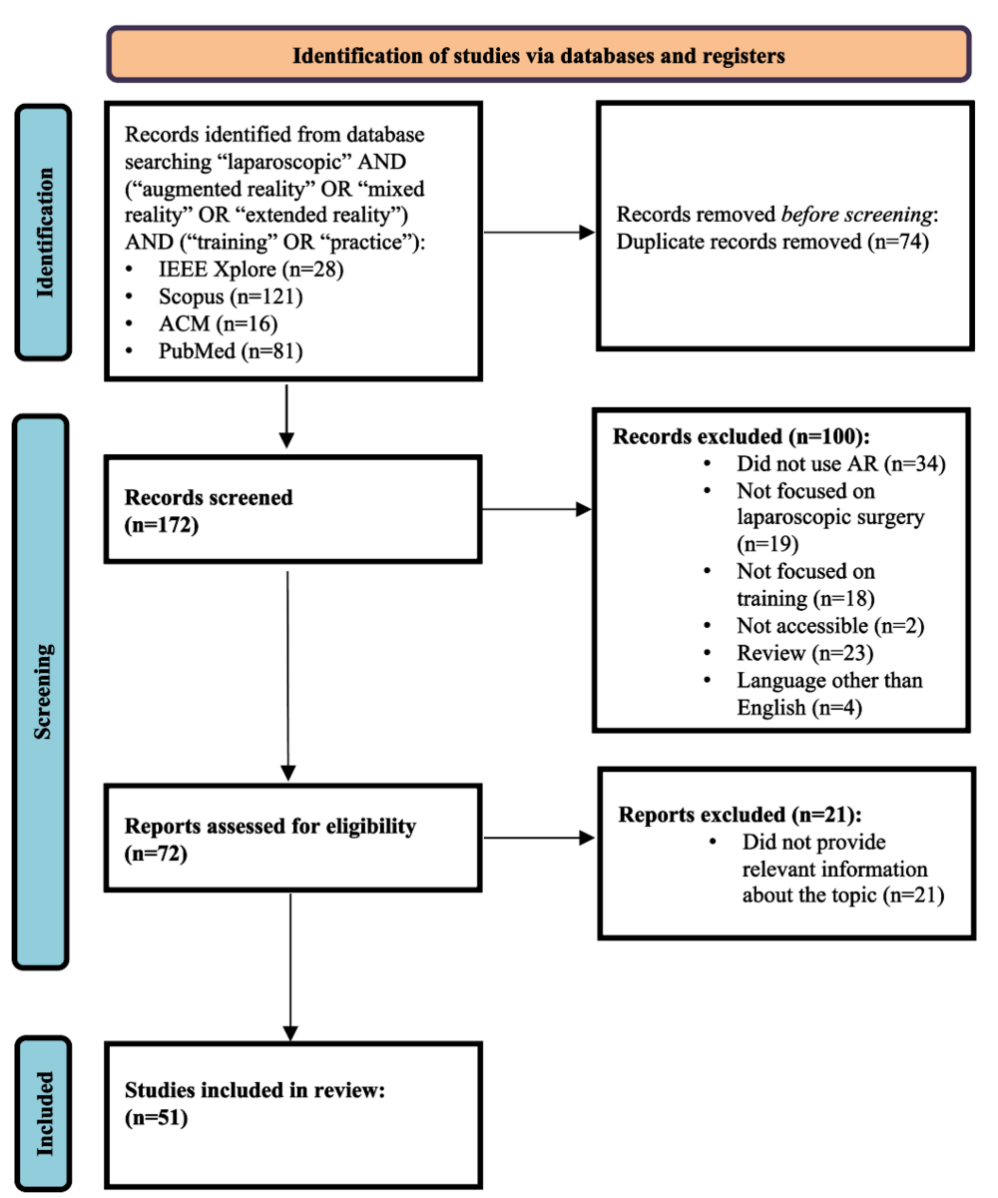
PRISMA-ScR (Preferred Reporting Items for Systematic Reviews and Meta-Analyses extension for Scoping Reviews) flow diagram showing the manuscript selection process. AR: augmented reality.

A total of 246 records were obtained (n=28, 11.4% in IEEE Explore; n=121, 49.2% in Scopus; n=16, 6.5% in ACM; and n=81, 32.9% in PubMed). After removing duplicate records, 69.9% (172/246) remained. Once the exclusion criteria were applied, 44.2% (76/172) of the articles were left. Of these 76 articles, 25 (33%) that did not meet the quality criteria were eliminated so that 51 (67%) articles were finally included in this review Figure 2 illustrates the complete workflow.

The papers included were published between 2002 and 2023, most of them (37/51, 73%) were scientific journal articles [6,8-43], and the rest (14/51, 27%) were papers presented in scientific conferences [44-57].

The evolution of the papers included in this scoping review was heterogeneous (Table 1), although it seems to be related to the launch of new AR devices on the market. Among them, the appearance of the first AR applications in games and smartphones in 2009, the launch of the Google Glass device in 2015, or the launch of the second version of Microsoft’s HoloLens in 2019 are highlighted.

In the following sections, we will analyze each of the RQs defined to provide a comprehensive analysis of the current application of AR technology in laparoscopic surgery training.

**Table 1:** Article distribution by year (N=51).

Year	Articles, n (%)
2002	1 (2)
2005	1 (2)
2006	1 (2)
2007	1 (2)
2008	2 (4)
2009	3 (6)
2010	4 (8)
2011	3 (6)
2012	3 (6)
2013	3 (6)
2014	6 (12)
2015	1 (2)
2016	2 (4)
2017	2 (4)
2018	1 (2)
2019	2 (4)
2020	3 (6)
2021	4 (8)
2022	4 (8)
2023	4 (8)

### RQ 1: What Types of Devices and Feedback Are Used for AR-Based Laparoscopic Surgery Training?

Our first RQ was about the AR devices used and the information provided. This analysis allowed us to see the evolution of the technology used and how these systems provide relevant information to enhance laparoscopic surgery training.

Regarding the devices, we classified them into 2 main types: optical see-through (OST) and AR video (ARV)–based devices (Table 2). OST refers to those devices that are capable of rendering information on a medium while allowing the real world to be seen through it. Devices such as HoloLens fall into this category as they allow information to be rendered on the screen while superimposing this information on our visual perception of the real world. ARV refers to the use of conventional video devices such as monitors, tablets, or other types of screens in which reality information is displayed (such as a real-time video) and overlaid with expanded information, creating an AR system. OST systems usually include voice commands, eye tracking, and interaction with real-world objects, whereas ARV systems reduce interaction and rely mainly on the display of additional information on the screen. Some articles (2/51, 4%) did not specify the device used, and so they were classified as unspecified devices.

The feedback provided by the system is closely related to the devices being used as it conditions the user-system interaction. We divided the articles by the information given to the user, differentiating among (1) *execution-related* information, which refers to any type of information related to the actions being performed by the trainee, such as the force exerted or time expended; (2) *mentor guidance*, which refers to those systems in which the AR technology allows the mentor to enhance the way in which they provide guidance to the trainee, for instance, using virtual pointers or annotations; (3) *educational information*, which could be general information about the training activity being performed, such as images of the organs and structures involved in the surgical activity or other general information; and, finally, (4) *patient-oriented* information, which corresponds to personalized information about the patient to be operated on. In this case, information was divided into *medical imaging* (as preoperative imaging), *3D segmentation* created from preoperative imaging, or *other* personalized information. *Other information* comprised information that did not fit into any other classification. *Unspecified information* comprised articles that did not indicate the type of information provided to the user of the AR device (Table 2).

**Table 2:** Type of information provided to the user of augmented reality–based laparoscopic training systems.

	Execution related	Mentor guidance	Educational information	Patient-oriented information			Other information	Unspecified information
				3D segmentation	Medical imaging	Others		
ARV^a^	Zahiri et al [14] Nomura et al [15] Horeman et al [21,26] Botden et al [34,35] Preetha et al [44] Lahanas et al [45] Rozenblit et al [46]	Wild et al [8] Andersen et al [16] Vera et al [19] Felinska et al [41] Cizmic et al [42] Shabir et al [47]	Arts et al [12]	Pessaux et al [20] Condino et al [48] Fusaglia et al [49]	Viglialoro et al [13] Teber et al [33] Koehl et al [50]	Shao et al [43] Viglialoro et al [51]	Hughes-Hallett et al [17] Lahanas et al [18] Loukas et al [22] Baumhauer et al [38] Kim et al [39] Ivanova et al [52] Wagner and Rozenblit [53] Larrarte and Alban [54]	Nugent et al [23] Brinkman et al [24] Pagador et al [25] Strickland et al [28] Leblanc et al [29-32] Botden et al [36,37] Van Sickle et al [40]
OST^b^	Cau et al [9]	—^c^	Simone et al [10] Doughty et al [55]	Sánchez-Margallo et al [6]	Sánchez-Margallo et al [6] Simone et al [10] Zorzal et al [11]	—	Rewkowski et al [56]	—
Unspecified device	—	—	—	—	—	—	—	Pagador et al [27] Gupta et al [57]

^a^ARV: augmented reality video.

^b^OST: optical see-through.

^c^Combinations of devices and information types under which no articles were categorized, indicating nonapplicability in the reviewed studies.

Most of the studies analyzed (43/51, 84%) used ARV devices, and only 12% (6/51) used OST devices. A total of 4% (2/51) of the articles did not specify which kind of device was used.

Regarding the ARV studies, although most of them (11/43, 26%) did not specify what information they provided to the user, among those that specified this information, execution-related information was highly common (9/43, 21%), such as in the study by Zahiri et al [14], which showed the time remaining for the trainee to complete the current task in the form of either a numerical timer or a progress bar. Regarding mentor guidance (6/43, 14%), Andersen et al [16] developed a tool for tele-mentoring, allowing the mentor to use different-colored dots for annotation. A common type of information was patient-oriented information (8/43, 19%). Pessaux et al [20] conducted a 3D segmentation from preoperative images to generate a 3D model of the patient’s body, which was later superimposed over the real patient’s body during a duodenopancreatectomy. Regarding medical imaging, Koehl et al [50] developed a system that allowed trainees to visualize and manipulate preoperative images. Arts et al [12] provided trainees with educational videos with instructions for the ongoing task. Regarding other information, Shao et al [43] used prerecorded ultrasound videos that were played depending on the position of the laparoscopic tools so that their system could be used for training purposes on ultrasound-guided laparoscopic interventions.

Of the 6 studies using OST devices, 3 (50%) used HoloLens version 1 [10,11,56], and 3 (50%) used HoloLens version 2 [6,9,55]. In addition, the year of publication of those articles was 2020, so it could be considered a technology of recent application in laparoscopic training. Although far fewer studies that used OST devices were found, they mainly provided patient-oriented information (4/6, 67%). For instance, Zorzal et al [11] gave users the possibility to obtain access to the preoperative magnetic resonance image of the patient. Others (2/6, 33%) provided educational information, such as the study by Simone et al [10], which used annotations on the patient’s body and a mentor’s voice instructions.

### RQ 2: What Type of Sensorization Is Used for AR-Based Laparoscopic Surgery Training?

To assist in the training activity, it is essential to know in real time the location of the different elements involved in the training environment, such as the training scenario, surgical instruments, patient, and posture of the surgeon. By monitoring these elements, it is possible to adapt the training process to the trainee’s educational needs. In addition, as this task is manipulative, it is relevant to consider the haptic stimuli. Therefore, in this section, we analyze which aspects related to the use of sensors (sensorization) were used in scientific literature. In this regard, we classified systems according to 2 aspects: the element being measured or sensorized (Table 3) and the technology used (Table 4).

**Table 3:** Classification by type of element being measured and technology.

Element being measured or sensorized		Studies
**User tracking**		
	Force feedback	[26,39,46,52]
	Body	[6,8,29,42]
	Eye	[11,41]
	Instrument	[9,12,15,18,19,21-25,27-40,43,45,46,49,51-54]
**Target tracking**		
	Body	[13,14,16,17,20,22,33,38,48,49,51,53]
	Instrument	[13,44,56]
Unspecified		[10,47,50,55,57]

**Table 4:** Classification of articles by sensorization technology.

Technology	Studies
Sensor based	[6,11,13,18,21,25,27,33,51]
Optic	[8,9,12-20,22-24,28-33,35-38,40-45,48,49,53,54,56]
Force	[26,34,39,46,52]
Unspecified	[10,47,50,55,57]

As we have pointed out, there are different elements in the training environment that can be tracked to know their location, motion, or behavior. We distinguished *user tracking* (ie, monitoring the surgeon and elements related to the surgeon). In this case, we organized the tracked elements into 3 categories: *body*, which were systems that recorded the surgeon’s body motion for kinematic analysis; *eye*, to refer to the analysis of the surgeon’s gaze and where they focus their attention; and *instrument*, which referred to the tracking of the surgical instruments. In addition to the user, the target can be tracked (*target tracking*), that is, the patient or the simulated model of the patient. This type of analysis was usually conducted using computer vision techniques. In this case, when we talk about *body*, we refer to the patient’s body and organs, whereas *instrument* refers to other objects or markers that can be placed inside the patient and can be tracked or recognized, such as the needle used in suturing tasks [44].

Not only the object being tracked is important but also how they were tracked, that is, the technology and techniques used to achieve this tracking. For this reason, we divided the technology into 3 categories: *sensor based*, *optic*, and *force*. *Sensor-based* technology refers to those systems that used a set of sensors specifically located to track and measure certain interactions in those locations. An example could be the use of electromagnetic trackers for recording laparoscopic tool motion [25]. *Optic* technologies refer to the identification and tracking of objects using artificial vision techniques. Felinska et al [41] used iSurgeon, an AR tool that allowed instructors to project their hand gestures in real time onto the laparoscopic screen, enhancing mentoring guidance during laparoscopic training sessions. *Force* refers to those systems that used devices capable of measuring the interaction force of the laparoscopic instrument [26].

Most of the studies (46/51, 90%) used some type of sensorization of the training environment in which the surgical simulation was performed.

In most AR systems, the user saw a real world augmented only with visual information and had no means to interact with virtual objects. If we want to manipulate virtual objects, we need another kind of information—*haptic* [58]. Therefore, together with the display of visual information recorded by tracking devices, the availability of augmented haptic information seems relevant in laparoscopic surgery training, where manipulative tasks are fundamental. In this sense, 8% (4/51) of the studies included some type of haptic feedback in the laparoscopic surgery training environment. For example, Ivanova et al [52] used a virtual 3D model of an organ and a sensorized physical simulator, providing force feedback on the hardness of the tissue being touched and the force exerted.

Regarding the element being measured or sensorized (tracked), the most common was tracking of the surgeon (user tracking). In this case, the most relevant elements tracked were the instruments used by the surgeon (32/51, 63%). Pagador et al [27] used electromagnetic trackers to record the instrument’s position during the training activity, whereas Lahanas et al [45] used fiducial markers attached to the tip of the instrument for tracking instrument position and rotation. There were only a few studies (4/51, 8%) that made use of surgeon posture tracking. For example, Cizmic et al [42] tracked hand position to project the hand movements of the trainer onto the laparoscopic image to enhance communication between trainer and trainee. The use of eye tracking was present in only 4% (2/51) of the studies analyzed. This technique was used by some authors to interact with sets of images and other elements of a user interface by means of gaze [11] or to assess performance and quality of communication [41].

Target tracking was the next most used sensorization in laparoscopic surgery training solutions. In this case, body tracking (tracking objects in the training environment or patient organs) was the category that comprised most of the reviewed articles. For this purpose, most of the studies (35/51, 69%) used artificial vision techniques to identify and track body structures. Viglialoro et al [13] used AR as a training aid in simulator-based laparoscopic cholecystectomy training, mainly during the isolation of the cystic duct and artery (Calot triangle). AR technology allowed trainees to visualize these hidden structures during the training activity [13]. Pessaux et al [20] proposed an AR-based assistance system to superimpose information from preoperative images onto the patient’s skin using a beamer, generating body transparency with visualization of deep structures. Finally, only few studies (2/48, 4%) tracked information about artificial markers or objects (instruments) within the surgical workspace inside the patient’s body. Preetha et al [44] tracked the surgical needle during a laparoscopic suturing task.

### RQ 3: What Type of Simulator and Setup Is Used for AR-Based Laparoscopic Surgery Training?

Regarding the simulator and setup used for laparoscopic training, 2 main characteristics were considered. The first was the origin of the training system, which may be a widely used commercial simulator (eg, ProMIS) or a prototype developed specifically for the research being carried out or pending to be released into the market. The second was the physical characteristics of the simulator and the models used in the training activity (training model). It could be an artificial model created solely for the purpose of training or an organic model (in vivo or ex vivo) obtained from organic tissue (Table 5).

**Table 5:** Classification of the different training models (artificial training model, ex vivo, and in vivo) according to the type of simulator (commercial or prototype).

		Training model: artificial	Organic			Unspecified
			Ex vivo	In vivo		

Prototype		Viglialoro et al [13] Andersen et al [16] Horeman et al [21,26] Kim et al [39] Shao et al [43] Shabir et al [47] Condino et al [48] Viglialoro et al [51] Wagner and Rozenblit [53]	Baumhauer et al [38]	—^a^	Zorzal et al [11] Loukas et al [22] Rozenblit et al [46]	
**Commercial**						
	ProMIS	Nomura et al [15] Nugent et al [23] Brinkman et al [24] Botden et al [34,35] Van Sickle et al [40]	Strickland et al [28] Leblanc et al [31,32]	—	Leblanc et al [29,30] Botden et al [36,37]	
	FLS^b^	Zahiri et al [14] Vera et al [19]	—	—	—	
	Others	Wild et al [8] Arts et al [12]	Pagador et al [25] Cizmic et al [42] Fusaglia et al [49]	—	—	
Unspecified		Cau et al [9] Lahanas et al [18] Preetha et al [44] Lahanas et al [45] Larrarte and Alban [54] Doughty et al [55] Rewkowski et al [56]	Felinska et al [41] Ivanova et al [52]	Sánchez-Margallo et al [6] Simone et al [10] Pessaux et al [20] Teber et al [33]	Hughes-Hallett et al [17] Pagador et al [27] Koehl et al [50] Gupta et al [57]	

^a^Combinations of devices and information types under which no articles were categorized, indicating nonapplicability in the reviewed studies.

^b^FLS: Fundamentals of Laparoscopic Surgery.

The use of commercial simulators was more widespread (20/51, 39%) than the use of prototypes (14/51, 27%). Commercial simulators mostly used artificial models (10/20, 50%), such as beads for the eye-hand coordination tasks [15]. There were also some studies (6/20, 30%) that used organic models. In this case, some authors compared skill acquisition after training with human cadavers versus using AR-enhanced artificial models [31]. Strickland et al [28] used a lamb liver to which a piece of marshmallow was introduced to simulate a tumor to be removed, and Pagador et al [25] used a porcine stomach for suturing tasks. There were some studies (4/20, 20%) that did not specify the model used by the commercial simulator or that could not be included in any of the defined categories.

Regarding the simulator prototypes, most (8/12, 67%) used artificial models. Lahanas et al [18] used markers inside a box trainer to superimpose images of different interactive objects, such as rings or beads that needed to be manipulated. Regarding the use of organic models, only Baumhauer et al [38] used an ex vivo porcine kidney to test the image overlay capability of the AR-based training system presented. The rest of the papers did not specify the model used or did not fit into any of the categories.

Artificial models were the most used training models (26/51, 51%), especially in simulator prototypes (10/17, 59%), followed by ex vivo training models (8/51, 16%). The latter stand out as 62% (5/8) of the studies that used ex vivo models used commercial simulators. The studies that used in vivo models did not refer to the simulator used as these were real operations (either on humans or animals).

There were studies that did not specify whether they used a commercial simulator or developed a prototype (17/51, 33%). Of these studies, 41% (7/17) used artificial training models, such as the system presented by Doughty et al [55], which did not use a specific laparoscopic simulator as it was intended to be used in any type of surgery. Meanwhile, 6 of the studies that did not specify whether they used a commercial simulator or a prototype used organic models, with 4 (67%) of them being in vivo models. Simone et al [10] used MR smart glasses to enable tele-mentoring in real surgical procedures during the COVID-19 pandemic.

There were a few articles (4/51, 8%) that did not specify either the type of simulator or training model used. Gupta et al [57] mentioned the need for a development framework for this type of simulators in laparoscopic surgery training and which aspects should be considered. However, they did not make use of any training system as it was a theoretical study [57]. Another study tested the impact of AR elements on inattentional blindness during a laparoscopic operation using a prerecorded video [17]. The study by Koehl et al [50] focused on the generation of modular models that could be used for other applications as a 3D visual model or as a force feedback generation system. Pagador et al [27] designed and evaluated a laparoscopic tool tracking system for further assessment purposes.

### RQ 4: What Type of Evaluation Is Used to Assess Skill Acquisition in AR-Based Laparoscopic Surgery Training?

In total, 2 main ways of addressing the assessment of laparoscopic skill acquisition in AR-based laparoscopic surgery training solutions were reported. One way of evaluation was through objective evaluation metrics [15-18,22-26,28-30,32-34,38,40-42,44,49,52,54-56] usually calculated by the training system. Another way was subjective evaluation carried out by experts or by means of interviews or self-assessment questionnaires [6,10,11,36,43,47]. Some studies used both ways [8,9,12-14,19,21,31,35,37,48], while others used none [20,27,39,45,46,50,51,53,57].

Objective evaluation was the most used method for the assessment of skill acquisition in laparoscopic surgery. In terms of the metric used, most of the studies (25/51, 49%) evaluated the performance of surgeons to assess whether the system enhanced laparoscopic skill acquisition. A variety of metrics were used, such as execution time, path length, or motion smoothness (Table 6). Time, whether related to task completion or training duration, was a commonly used metric [41] that assessed the time spent on each task as a performance indicator. There were other studies (2/31, 6%) that, instead of measuring the time needed to complete the task, measured the time that the laparoscopic instruments spent in a specific area, for instance, during a suturing task [35].

**Table 6:** Type of metric used for objective evaluation in each study analyzed.

Metric	Studies
Training time or task time	[8,12,19,23,28,34,35,40,41]
Time in correct area	[34,35]
Path length	[15,18,19,21,24,25,29,30]
Objective Structured Assessment of Technical Skills	[8,31,32,41,42]
Global Operative Assessment of Laparoscopic Skills	[8,42]
Economy of movement	[15,24]
Force	[21,26,34,35]

Path length was another prevalent objective metric (8/31, 26%). This metric records the path followed by the laparoscopic instruments during the performance of the training task. There were another 6% (2/31) of the studies that, apart from measuring path length, also computed the economy of movements of the surgical instruments [15,24]. Force metrics were also commonly used (4/31, 13%). Botden et al [34] evaluated the performance of a suturing task by assessing the strength of the knot. The Objective Structured Assessment of Technical Skills (OSATS) and the Global Operative Assessment of Laparoscopic Skills (GOALS) formularies are 2 recognized standards for assessing laparoscopic skills that were also used in some of the studies, with the OSATS being used in 14% (5/36) of the studies and the GOALS being used only twice (2/36, 6%).

Subjective evaluation was mostly used for evaluating the usability of the AR tools (6/51, 12%). For example, Arts et al [12] made use of a questionnaire to assess the validity of the ProMIS as an AR laparoscopic surgery simulator. The combination of both types of evaluation (objective and subjective) was used in 22% (11/51) of the studies. Cau et al [9] combined an expert’s subjective assessment of a suturing task with metrics obtained by the system itself.

### RQ 5: What Types of Surgical Tasks or Procedures Are Used in AR-Based Laparoscopic Surgery Training?

Regarding the laparoscopic training activities (tasks or procedures), in most of the studies (26/51, 51%), trainees performed basic tasks commonly used in the early stages of laparoscopic surgery training programs. These tasks were divided into 5 main categories: navigation tasks focused on the skill needed to explore the surgical workspace using the camera and laparoscopic instruments; object manipulation tasks aimed to train the user to move, rotate, or manipulate objects or organs using the laparoscopic instruments; dissection tasks focused on the ability to separate tissue and anatomical structures without causing any damage to them; cutting tasks focused on training users to make precise cuts in organs and tissues; and suturing tasks focused on training for suturing tissues, including knotting skills. Finally, there were studies that used surgical procedures such as partial nephrectomy on ex vivo models [38] or sigmoid colectomy on cadavers [31].

Similarly, considering its main clinical features, such as the surgical procedure performed or the training model used, the category of surgical specialty into which each study fell or could fall was defined. For those studies in which a training surgical intervention was performed, the surgical specialty was the one which that procedure corresponded to. There were also some studies that included simple tasks. In this case, the surgical specialty was selected based on the training model used. For those cases in which surgical skills did not focus on a particular surgical specialty, the studies were classified as “All” specialties (Table 7).

**Table 7:** Studies organized by type of training task or procedure and surgical specialty.

	Navigation	Object manipulation	Dissection	Cutting	Suturing	Surgical procedure	Unspecified
General	Rozenblit et al [46]	Rozenblit et al [46]	—^a^	—	—	Wild et al [8] Simone et al [10] Viglialoro et al [13] Pessaux et al [20] Strickland et al [28] Leblanc et al [29-32] Felinska et al [41] Cizmic et al [42] Shao et al [43] Fusaglia et al [49] Viglialoro et al [51]	Ivanova et al [52]
Urology	—	—	—	—	—	Sánchez-Margallo et al [6] Simone et al [10] Teber et al [33] Baumhauer et al [38]	—
Thoracic	Loukas et al [22]	—	—	—	—	—	—
All	Lahanas et al [45] Wagner and Rozenblit [53]	Zahiri et al [14] Nomura et al [15] Andersen et al [16] Horeman et al [21] Nugent et al [23] Botden et al [36] Rewkowski et al [56]	Arts et al [12] Nugent et al [23] Brinkman et al [24]	Arts et al [12] Andersen et al [16] Brinkman et al [24]	Cau et al [9] Vera et al [19] Pagador et al [25] Horeman et al [26] Botden et al [34-36] Van Sickle et al [40] Preetha et al [44] Shabir et al [47]	Zorzal et al [11] Koehl et al [50] Doughty et al [55]	Pagador et al [27] Condino et al [48]
Unspecified	Larrarte and Alban [54]	Lahanas et al [18]	—	Lahanas et al [18]	Botden et al [37]	—	Hughes-Hallett et al [17] Kim et al [39] Gupta et al [57]

^a^Combinations of training task or procedure and clinical specialty under which no articles were categorized, indicating nonapplicability in the reviewed studies.

A large proportion of the studies analyzed (20/51, 39%) used surgical procedures rather than simple tasks to train and assess surgical skills. General surgery was the surgical specialty of most of the studies (14/20, 70%). In the study by Viglialoro et al [13], they presented an AR solution that assisted in the identification of the artery and cystic duct in a training simulator for cholecystectomy. Other studies (4/20, 20%) were included under the specialty of urology, such as the study by Teber et al [33], who presented a system for formative assistance during laparoscopic partial nephrectomy. Other studies (3/20, 15%) performed procedures that were not classified into any specific surgical specialty but could be used in any specialty, such as the study by Doughty et al [55], who developed a context-aware system capable of assisting the surgeon depending on the training task being performed.

Regarding basic laparoscopic surgery training tasks, the most common task used was suturing (11/25, 44%). Botden et al [34], for example, used suturing to analyze the evolution of the trainees’ technical skills, assessing how fast trainees performed tasks and the strength of the knotting. Object manipulation tasks was also common in the studies analyzed (9/25, 36%). Lahanas et al [18] presented 3 different object manipulation tasks to assess trainees’ laparoscopic skills using their AR-based simulator. The use of navigation tasks was less common in the training settings (5/25, 20%). Fusaglia et al [49] presented an overlay system to show hidden anatomical structures during the performance of laparoscopic navigation tasks. The first task was to press a set of buttons in a specific order using the laparoscopic instruments, the second task was to transfer an object using the laparoscopic tools, and the last task was to cut a virtual object (using AR technology). Another type of task were dissection tasks, but they were less common that the rest (3/25, 12%). One study proposed the use of 2 balloons (one inside the other) that the trainee had to separate keeping the inner balloon inflated (ie, without being damaged) [12]. Only 16% (4/25) of the studies focused on cutting tasks. In the study by Brinkman et al [24], a full week of laparoscopic surgery training was proposed, having training sessions once a day in which some laparoscopic cutting tasks were included. Finally, there were 12% (6/51) of the articles that did not specify the task performed.

## Discussion

### Principal Findings

#### Overview

AR is an emerging technology that is being applied to various fields, including health care. In this sense, applications have been developed mainly oriented toward surgical planning, medical and surgical training, and surgical assistance [59,60]. In this study, a review of scientific literature oriented toward solutions for assistance in laparoscopic surgery training was carried out. We analyzed aspects that we considered critical to innovate and advance in this field, such as the devices used to provide AR to the user, the training environments, the types of training tasks or procedures, and the evaluation metrics used to analyze the evolution of students during the training activities and provide them with educational feedback. For this purpose, we analyzed all the RQs raised in this work with the aim to provide some observations that may help researchers guide their new proposals and innovative solutions to achieve an improvement in the field of laparoscopic surgery training.

The evolution of the number of publications included in this scoping review was heterogeneous and may be associated with the evolution of AR technology and the introduction of new applications and devices. Although there were some studies in the first years of this century associated with the appearance of the first AR applications in games and smartphones, it was in 2009 when a relevant increase in interest in the use of AR was observed, coinciding with the success of applications such as the AR toolkit, an open-source library for the creation of AR applications based on the identification of visual markers. This relevant interest continued until 2015, which could be related to the launch of Google Glass. This device was the first proposal that took AR out of the screen and into the real world using a wearable device. However, it was not until the launch of the second version of Microsoft’s HoloLens (2019) when the potential of this type of technological proposals increasingly distanced from solutions linked to screens began to be glimpsed. This fact may be related to the recent increase in interest in the use of this type of AR-linked technologies in laparoscopic surgery training.

In the following sections, we will analyze the results to identify relevant insights that address each of the RQs.

#### Devices and Feedback (RQ 1)

While video devices such as monitors, laptops, smartphones, and tablets have traditionally been used in the early stages of AR technologies to enhance laparoscopic surgery training, the use of OST devices is a relatively new development due to the recent emergence of devices that facilitate these functions (the first device appeared in 2020). Therefore, only 12% (6/51) of studies were found that explored the application of OST technology in this context [6,9-11,55,56].

OST devices offer greater versatility than traditional video devices (monitors, smartphones, and tablets) for laparoscopic surgery training. As several studies (6/51, 12%) demonstrated, OST devices provide novel interaction methods and functionalities such as eye or gaze tracking, voice commands, and gestural interactions to manipulate virtual objects or add annotations. For instance, the head gaze was used to point at the laparoscopic virtual screen to enhance communication between trainer and trainee [11]. Felinska et al [41] used eye-tracking technology to create a heat map and analyze differences between the visual focus areas of trainees and instructors. In another study using OST technology, the Vuforia library was used to recognize artificial visual markers in the training environment to insert virtual elements to enhance the experience of laparoscopic surgery training [56]. However, this solution was limited to simulated environments, making it difficult to use in real clinical settings as it relied on 5 cameras for marker recognition. Sánchez-Margallo et al [6] and Simone et al [10] focused on the creation of 3D models from real preoperative images to implement AR-based training applications. In addition, this functionality was extended by allowing tutors to add annotations and information to the models [10].

One of the advantages of OST devices is that they can be used in any training environment used in laparoscopic surgery. The trainee always visualizes the real training environment, and it is the augmented information (holograms) that is superimposed onto the real image through the glasses. For instance, in the study by Sánchez-Margallo et al [6], it was not necessary to connect the OST device to the laparoscopic simulator as it did not require the laparoscopic video feed.

However, Zorzal et al [11] accessed the video source from the laparoscopic tower and explored the ergonomic improvements that OST devices could provide by displaying the endoscopic video in any place of the operating room and even following the surgeon’s head.

Regarding ARV devices, the analyzed studies highlighted their potential to enhance the experience during laparoscopic surgery training. In the study by Preetha et al [44], a convolutional neural network was used to predict depth from laparoscopic imaging and recognize the manipulation of laparoscopic instruments for use in surgical skill assessment. Another study also used depth prediction to generate and display optimal trajectories for surgical instruments [53]. Solutions for depth perception support were also presented to improve the training process of novice trainees in laparoscopic surgery [45,49]. The presence of assistive information in the surgeon’s field of view is not a major disadvantage as it has been shown that the use of information-enhanced holograms does not have a negative impact on the surgical working field [17]. In another case, ARV technology was used to generate 3D models from preoperative imaging and superimpose them onto laparoscopic images as a support during the performance of the surgical task or formative procedure [20].

Another important aspect to consider during laparoscopic surgery training is to analyze force feedback during the performance of different laparoscopic tasks and procedures. This factor was explored in an ARV training environment, showing that feedback of force exerted improves the acquisition of tissue-handling skills [21,26]. Other studies (6/51, 12%) investigated the overlay of assistive content in the performance of training tasks and procedures by means of ARV applications and concluded that it helps the trainees reduce path length and deviation along the depth axis and improve their orientation skills [18,48,49,52-54].

All in all, we can see that both OST and ARV devices show significant potential as training aids in laparoscopic surgery. Although OST devices are a more recent and less explored development, they offer enhanced versatility compared to traditional video devices used in early AR technologies. The reviewed studies highlight the various applications of OST and ARV, including novel interaction methods, access to preoperative imaging, ergonomic improvements, depth analysis, and instrument motion analysis. This suggests that both OST and ARV have the potential to enhance laparoscopic surgery training, with OST devices offering greater versatility and opportunities for innovation in this field.

#### Sensorization (RQ 2)

The studies analyzed in this review mainly presented 2 types of sensing technologies: haptics and tracking. The use of haptic technology in laparoscopic surgery training has been a topic of considerable interest, as evidenced by the number of studies found on this topic (9/51, 18% of the studies in total). These studies explored the potential benefits of haptic feedback in enhancing the acquisition of laparoscopic skills and improving surgical performance. The use of haptic feedback in laparoscopic surgery simulation was examined in several studies (4/51, 8%), demonstrating its efficacy in improving surgeons’ dexterity [24,26,39,52] The incorporation of haptic feedback in AR-based laparoscopic surgery training applications has been shown to lead to improved performance among novice surgeons [61] and improve surgeons’ skills in laparoscopic suturing [23]. The efficacy of haptic feedback in laparoscopic skill acquisition was also studied by other authors (4/51, 8%) [34,36,37,62], who showed that it significantly enhanced surgeons’ precision and instrument manipulation skills. These studies highlighted the potential of haptic feedback as a valuable adjunct to laparoscopic surgery training. It seems evident that haptic technology promises further advances in laparoscopic surgery training and proficiency.

Regarding tracking technologies, there were 2 main approaches: a sensor-based approach and an optical technology–based approach. The sensor-based approach uses sensors placed at specific locations to track the object in the training environment. Meanwhile, the optical technology–based approach uses image analysis to perform tracking of the objects of interest. The studies included in this review used artificial vision techniques and optical sensorization, mainly by using the laparoscopic camera feed. For instance, Loukas et al [22] used 2 different algorithms to estimate the tooltip position, one of them using an adaptive model of a color strip attached to the tool and the other one tracking directly the shaft of the tool. Optical technology plays a crucial role in accurate tracking in clinical settings. Zahiri et al [14] used this technology to evaluate the performance of an object transfer task in basic training environments by tracking colored rings. Another study focused on tracking anatomical structures such as the Calot triangle and user interaction during the training procedure [13]. Vision-matching techniques enhance trainees’ visualization of hidden anatomical structures by superimposing 3D models onto the laparoscopic camera source, improving trainees’ understanding of spatial relationships [33]. Vision-matching techniques were also used for pairing of real and augmented vision, some of them automatically and others semiautomatically. Thus, in the study by Hughes-Hallet et al [17], a semiautomated real-time overlay of preoperative models onto the anatomical field structures was performed, improving accuracy and efficiency in the AR-guided training process, whereas Pessaux et al [20] and Condino et al [48] used manual assistance to align the virtual objects with the patient’s anatomy. Overall, the integration of artificial vision techniques and optical technology plays a crucial role in the identification and tracking of the anatomical structures of the patient. These solutions improve the understanding of the steps to be carried out during training activities, as well as the identification of manipulated objects by trainees in laparoscopic surgery training.

The eye-tracking technology present in many of the OST devices has shown great potential in various fields, including human-computer interaction and cognitive sciences. However, in the context of laparoscopic surgery training, the use of this technology remains relatively unexplored. In this scoping review, only 4% (2/51) of the studies investigated the incorporation of eye-tracking technology for assistance in laparoscopic surgery training. In one of the studies, gaze tracking (using head tracking) was performed to focus the laparoscopic camera on the area where the surgeon’s gaze was centered [11]. This study made it possible to optimize the way of visualizing the information on the surgical working environment and the surgeon’s ergonomics. Another study aimed to use eye-tracking technology to detect convergence between the trainee’s and the trainer’s gaze, as well as between the trainee and the target area [41]. By monitoring eye movements and fixation points, the system could provide objective feedback on the trainee’s visual attention and alignment with the desired gaze targets. This approach has the potential to enhance trainee-trainer communication and improve the trainee’s spatial awareness within the surgical field. Considering these aspects, there is a clear need for further investigation on the use of this technology to improve the training process in laparoscopic surgery. Eye tracking offers unique advantages, such as providing the trainee’s visual focus, gaze patterns, and cognitive workload in real time. The integration of eye-tracking technology into laparoscopic simulators and training platforms can enhance the visual perception, spatial orientation, and overall surgical performance of the trainee.

In addition, tracking of laparoscopic instruments and deformations in bodies or organs was also performed using artificial visual markers. In some cases, these were colored markers that were placed around the distal area of the instrument shaft [22,37,40]. Reflective markers were also used, although in this case, it was necessary to use infrared light and camera filters for identification [9]. The use of these techniques helps improve the identification and tracking of the laparoscopic instruments, which is essential to evaluate the surgical performance of trainees and assist them during the training activities. In addition, various studies (3/33, 9%) explored the calculation and modeling of deformations in bodies or organs to be used in advanced AR applications for laparoscopic surgery training. In one study, visual markers were inserted directly on the liver so that they could be identified by the laparoscopic image and allow for the localization and superimposition of preoperative 3D models of the liver and other relevant information on the endoscopic image [33,38]. Another study used retroreflective markers on the laparoscopic instruments and within the formative surgical field to facilitate the superimposition on the laparoscopic image of a preoperative 3D model [49].

Another type of technology used for tracking surgical instruments and organs involved in the formative activity was the use of electromagnetic sensors. Viglialoro et al [51] incorporated these sensors into the laparoscope and bile ducts or arterial tree of an artificial training model for real-time monitoring during the performance of simulator tasks. Electromagnetic sensors inserted into nitinol tubes representing the ducts and arterial trees helped infer possible deformations of the sensed tubular structures. Consequently, the virtual scene was updated in real time, augmenting the laparoscopic images with information on the real-time position of these anatomical structures.

By using the aforementioned techniques for the calculation of organ deformations and the inclusion of 3D models, these studies significantly contributed to the integration of virtual models into the laparoscopic surgery environments, enhancing surgical training scenarios in real time.

#### Training Simulator and Setup (RQ 3)

Regarding the type of simulator used for AR-based laparoscopic surgery training, most of them were box trainers to which an AR assistance system was added. Of note is the ProMIS simulator (Haptica, Inc), which was one of the first commercial simulators for laparoscopic surgery training with AR functionalities. This simulator included basic training tasks such as dissection and suturing but also more complex procedures such as liver resection and sigmoid colectomy. This is the AR-based simulator with the largest number of scientific publications. Apart from this simulator, we highlight other commercial options to which AR-based laparoscopic surgery training assistance systems were added, such as the Fundamentals of Laparoscopic Surgery simulator (Limbs & Things Ltd) [14,19], the Szabo Pelvic Trainer simulator (ID Trust Medical) together with the iSurgeon system [8,42], the eoSim simulator (eoSurgical) [12], the SIMULAP-IC05 simulator (Jesús Usón Minimally Invasive Surgery Centre) [25], or the Body Torso laparoscopic trainer simulator (Pharmabotics Ltd) [49].

Regarding the training model, the use of artificial models was most common because they are generally more accessible than organic models, although with less realism. However, more and more realistic artificial models have been achieved by simulating the anatomy and behavior of real tissues, as in the case of the study by Viglialoro et al [51], in which they presented a 3D replica of the patient’s own liver and gallbladder. Other options presented were the enhancement of the physical model using AR imaging [18].

#### Evaluation (RQ 4)

In the different studies analyzed, various types of metrics were used to evaluate the quality of surgical performance and the surgical skills of trainees. These metrics, according to their nature, were divided into objective and subjective metrics.

Obtaining objective metrics is one of the main advantages of using AR techniques in laparoscopic surgery training. Some of the training simulators, as is the case of ProMIS, allow for recording the actions executed by the trainee to obtain metrics such as path length or motion smoothness of the laparoscopic instruments, which can help evaluate the trainee’s performance and learning curve without the need for constant supervision by an expert evaluator.

Although there are some metrics that were recurrently used in the analyzed studies, so far there is no standardized set of objective metrics used to evaluate performance or technical skills in laparoscopic surgery. The most common metrics used were execution time, path length, economy of movement, and motion smoothness of laparoscopic instruments [15,18,24,25,28-30,40]. Other metrics were also used for specific tasks, such as knotting strength, time spent in the correct suturing and knotting area [34,35], or force exerted by laparoscopic instruments on the target organ or tissue [21,26].

Other types of surgical performance quality assessments based on questionnaires administered by external evaluators were also used, such as the OSATS, a highly standardized surgical assessment tool [8,31,32,41,42]. Horeman et al [21] and Leblanc et al [31] also used similar questionnaires based on peer reviews to determine surgeons’ experience in laparoscopic surgery.

Subjective evaluation was commonly used for assessing the validity of the proposed solution. None of the studies used a standard questionnaire for assessing system validity, but many similarities were found in terms of the aspects of the systems they evaluated. For example, usability, realism, and didactic value were included in several studies (7/18, 39%) [12,13,19,35-37,48]. Questionnaires with Likert scales were the most commonly used format in the studies.

Regarding assessment, the size and type of the samples included in the studies were also important. There was a wide variation in sample size, with samples ranging from 10 individuals [13] to 270 participants [15]. Some studies (2/25, 8%) included >100 participants [12,15], but most of them (23/25, 92%) presented sample sizes of <100 participants [8,17,23,24,29,31,32,37,41,42] or <30 individuals [9,13,14,16,19,28,30,34,35,40,43,47,48].

Finally, the included studies encompassed participants with different levels of laparoscopic surgery experience: novices, intermediates, and experts. Novices, referring to individuals with limited or no previous laparoscopic surgery experience, were frequently included in the studies. Intermediate-level participants, characterized by a moderate level of experience, were present in a few studies (4/25, 16%) [12,28,36,43]. This may be because their inclusion makes it difficult to differentiate results between study groups as there is sometimes no clear difference between the intermediate group and the novice or expert groups. Experts, representing individuals with advanced laparoscopic skills, were also included in several studies (8/25, 32%). This stratification of participants allows for a comprehensive validation of the system’s effectiveness across different proficiency levels and helps detect the different needs that these groups may have.

#### Tasks and Procedures (RQ 5)

Regarding the tasks and procedures used as training activities in the studies analyzed, the most widespread ones were those focused on eye-hand coordination tasks, such as peg transfer, instrument movement, and instrument navigation tasks [14-16,18,21-24,36,45,46,53,54,56]. The next most used type of task was the simulator suturing task [9,19,25,26,34-37,40,44,47]. Although less frequent, some studies (17/51, 33%) also presented more complex models for procedures such as cholecystectomy [8,13,15,42,51]. In this case, models of the liver and biliary anatomy were used to facilitate the training of novice surgeons in gallbladder extraction using laparoscopic techniques. These types of procedures, although still basic, are highly appropriate for learning basic tasks such as the localization of critical anatomical structures (cystic duct and cystic artery) and the performance of dissection and cutting tasks for dissection and cutting of the cystic duct, cystic artery, and gallbladder removal. In a closer-to-reality environment, AR-based assistive tools were used for procedures such as cholecystectomy [41] in ex vivo (porcine) experimental models and sigmoid colectomy in human cadavers [31,32].

Considering that the main objective of AR-based training assistance applications in laparoscopic surgery is to provide the students with support tools during their training activities, the type of task or procedure to be chosen has a great influence on the usefulness of these tools. It seems evident that, in considerably basic tasks, the training assistance systems would not provide significant value to the student, mainly due to the low level of complexity of the activity. However, it is in the more challenging tasks and procedures where training assistance systems may provide remarkable training value. For instance, a task that can present a high level of difficulty for novice laparoscopic surgeons is the performance of suturing. Support tools can enhance reality for the student with visual information to assist in the performance of the suture, such as the proper grip of the needle, the passage of the needle through the tissue, or the process of double and single knotting. In more complex procedures such as laparoscopic cholecystectomy or lymphadenectomy, these training assistance systems could support the students in locating complex or hidden anatomical structures, as well as remotely transmitting instructions from tutors who are reviewing the students’ training process, among other possibilities.

### Limitations

Although the PRISMA-ScR guidelines were followed to carry out this scoping review to reduce possible biases, we must highlight some limitations encountered in our work. Although most relevant articles found in other databases such as Springer or Google Scholar also appeared in the databases consulted, it is possible that some papers were not included because these databases were not used directly. In addition, the specific search strategies and keywords used in the various databases may have led to the exclusion of articles that used different terminology to refer to the same topics. Although the number of articles affected was small, restricting the selection to English-language publications and excluding articles that we could not access may have led to the omission of studies that could have provided relevant information.

Regarding the devices used, OST devices are relatively new, so there is still much to explore in terms of what they can contribute to the field of laparoscopic surgery training, which we will address in future studies. It should also be noted that there may be interesting technical proposals for the support of laparoscopic surgery training that, as they have not yet been used for such training, were not included in the studies analyzed in our scoping review. Furthermore, the lack of standardized metrics for evaluating the different support systems did not allow for a comprehensive comparison between systems and a comparative analysis of their results.

### Conclusions

In conclusion, this scoping review sheds light on the dynamic landscape of AR technologies within laparoscopic surgery training. Although OST devices have recently emerged and their advantages over traditional AR methods are still being explored, it is relevant to indicate that the results obtained are promising and open up new opportunities for the use of AR in this type of training activities. In turn, haptic feedback emerges as a valuable asset for the acquisition of laparoscopic skills. Eye-tracking technology provides relevant information during learning, although its application is in an incipient phase that requires further exploration. The prevalence of commercial simulators and artificial models underscores the delicate balance between safety and realism. Regarding assessments, this review highlights the importance of extending the use of expert assessments, such as OSATS or GOALS, and underlines the need for standardized objective assessment. These assessments are based on the study of participants’ behavior in simple tasks such as navigation and object manipulation and in other tasks with a higher level of difficulty, such as suturing. In any case, it seems clear that AR would be more useful for more complex tasks related to complex surgical procedures. These findings beckon future research to analyze the complete potential of AR in enhancing laparoscopic skill acquisition.

## Appendix

Multimedia Appendix 1 Detailed search strategy.

Multimedia Appendix 2 PRISMA-ScR (Preferred Reporting Items for Systematic Reviews and Meta-Analyses extension for Scoping Reviews) checklist.

## Abbreviations

AR: augmented reality
ARV: augmented reality video
GOALS: Global Operative Assessment of Laparoscopic Skills
MR: mixed reality
OSATS: Objective Structured Assessment of Technical Skills
OST: optical see-through
PRISMA-ScR: Preferred Reporting Items for Systematic Reviews and Meta-Analyses extension for Scoping Reviews
RQ: research question
