# Supplementary material for: Use of Augmented Reality for Training Assistance in Laparoscopic Surgery: Scoping Literature Review
Source: J Med Internet Res. 2025 Jan 28;27:e58108. doi: 10.2196/58108 (PMC11815304; doi:10.2196/58108)
Supplement: Multimedia Appendix 1 [file jmir_v27i1e58108_app1.docx]

***Full search strategy***

**Scopus search query:**

( TITLE-ABS-KEY ( laparoscopic ) AND TITLE-ABS-KEY ( "augmented reality" OR "mixed reality" OR "extended reality" ) AND TITLE-ABS-KEY ( training OR practice ) ) AND ( LIMIT-TO ( DOCTYPE , "ar" ) OR LIMIT-TO ( DOCTYPE , "cp" ) ) AND ( LIMIT-TO ( LANGUAGE , "English" ) )

**IEEE Xplore search query:**

(("Document Title":laparoscopic OR "Abstract":laparoscopic OR "Author Keywords":laparoscopic OR "Index Terms":laparoscopic ) AND ( ("Document Title":training OR "Abstract":training OR "Author Keywords":training OR "Index Terms":training) OR ("Document Title":practice OR "Abstract":practice OR "Author Keywords":practice OR "Index Terms":practice) ) AND ( ("Document Title":"augmented reality" OR "Abstract":"augmented reality" OR "Author Keywords":"augmented reality" OR "Index Terms":"augmented reality") OR ("Document Title":"extended reality" OR "Abstract":"extended reality" OR "Author Keywords":"extended reality" OR "Index Terms":"extended reality") OR ("Document Title":"mixed reality" OR "Abstract":"mixed reality" OR "Author Keywords":"mixed reality" OR "Index Terms":"mixed reality") ))

**ACM search query:**

[[Title: laparoscopic] OR [Abstract: laparoscopic] OR [Keywords: laparoscopic]] AND [[Title: training] OR [Abstract: training] OR [Keywords: training] OR [Title: practice] OR [Abstract: practice] OR [Keywords: practice]] AND [[Title: augmented reality] OR [Abstract: augmented reality] OR [Keywords: augmented reality] OR [Title: mixed reality] OR [Abstract: mixed reality] OR [Keywords: mixed reality] OR [Title: extended reality] OR [Abstract: extended reality] OR [Keywords: extended reality]]

**Pubmed search query:**

(laparoscopic[Title/Abstract]) AND ( (training[Title/Abstract]) OR (practice[Title/Abstract]) ) AND ( (augmented reality[Title/Abstract]) OR (mixed reality[Title/Abstract]) OR (extended reality[Title/Abstract]) )
